# Individual Clinically Diagnosed with CHARGE Syndrome but with a Mutation in *KMT2D*, a Gene Associated with Kabuki Syndrome: A Case Report

**DOI:** 10.3389/fgene.2017.00210

**Published:** 2017-12-11

**Authors:** Sonoko Sakata, Satoshi Okada, Kohei Aoyama, Keiichi Hara, Chihiro Tani, Reiko Kagawa, Akari Utsunomiya-Nakamura, Shinichiro Miyagawa, Tsutomu Ogata, Haruo Mizuno, Masao Kobayashi

**Affiliations:** ^1^Department of Pediatrics, Hiroshima University Graduate School of Biomedical and Health Sciences, Hiroshima, Japan; ^2^Department of Pediatrics and Neonatology, Nagoya City University Graduate School of Medical Sciences, Nagoya, Japan; ^3^Department of Pediatrics, National Hospital Organization Kure Medical Center, Kure, Japan; ^4^Department of Diagnostic Radiology, Hiroshima University Graduate School of Biomedical and Health Science, Hiroshima, Japan; ^5^Miyagawa Kid’s Clinic, Hiroshima, Japan; ^6^Department of Pediatrics, Hamamatsu University School of Medicine, Hamamatsu, Japan; ^7^Department of Pediatrics, International University of Health and Welfare School of Medicine, Chiba, Japan

**Keywords:** CHARGE syndrome, Kabuki syndrome, *KMT2D*, *CHD7*, phenotypic overlap

## Abstract

We report a Japanese female patient presenting with classic features of CHARGE syndrome, including choanal atresia, growth and development retardation, ear malformations, genital anomalies, multiple endocrine deficiency, and unilateral facial nerve palsy. She was clinically diagnosed with typical CHARGE syndrome, but genetic analysis using the TruSight One Sequence Panel revealed a germline heterozygous mutation in *KMT2D* with no pathogenic *CHD7* alterations associated with CHARGE syndrome. Kabuki syndrome is a rare multisystem disorder characterized by five cardinal manifestations including typical facial features, skeletal anomalies, dermatoglyphic abnormalities, mild to moderate intellectual disability, and postnatal growth deficiency. Germline mutations in *KMT2D* underlie the molecular pathogenesis of 52–76% of patients with Kabuki syndrome. This is an instructive case that clearly represents a phenotypic overlap between Kabuki syndrome and CHARGE syndrome. It suggests the importance of considering the possibility of a diagnosis of Kabuki syndrome even if patients present with typical symptoms and meet diagnostic criteria of CHARGE syndrome. The case also emphasizes the impact of non-biased exhaustive genetic analysis by next-generation sequencing in the genetic diagnosis of rare congenital disorders with atypical manifestations.

## Introduction

CHARGE syndrome (OMIM #214800) is an autosomal dominant genetic disorder that was first reported in Pagon et al. (1981). Its characteristic features are coloboma, heart malformations, choanal atresia, growth and/or development retardation, genital anomalies, and ear malformations. Germline mutations in *CHD7* (OMIM ^∗^608892), encoding the chromodomain helicase DNA-binding protein 7 gene, have been identified in 58–67% of patients with CHARGE features (Vissers et al., 2004; Lalani et al., 2006; Zentner et al., 2010). Furthermore, the *CHD7* mutation detection rate rises to around 90% when patients meet the full CHARGE diagnostic criteria advocated by Blake or Verloes (Blake et al., 1998; Verloes, 2005; Jongmans et al., 2006; Janssen et al., 2012). However, *CHD7* mutations have also been identified in patients with Kallmann syndrome, idiopathic hypogonadotropic hypogonadism, autism spectrum disorder, and T cell immunodeficiencies such as complete DiGeorge syndrome and Omen-like syndrome (Ogata et al., 2006; Sanka et al., 2007; Gennery et al., 2008; Kim et al., 2008; Jiang et al., 2013). This suggests that germline *CHD7* mutations are associated with a broad clinical spectrum.

Kabuki syndrome (KS) (OMIM #147920 and #300867) is a rare multiple malformation disorder that was originally reported in Niikawa et al. (1981). Subsequently, the five cardinal manifestations of KS were defined by Niikawa et al. (1988) as typical facial features, skeletal anomalies, dermatoglyphic abnormalities, mild to moderate intellectual disability, and postnatal growth deficiency. However, consensus clinical diagnostic criteria for KS have not been established.

Two causative genes have thus far been identified in patients with KS (Ng et al., 2010; Jiang et al., 2013; Miyake et al., 2013). Germline mutations in *KMT2D* (OMIM ^∗^602113), encoding lysine-specific methyltransferase 2D, are responsible for the major molecular pathogenesis of KS, explaining the diagnosis of 52–76% of patients (Adam et al., 1993; Ng et al., 2010; Hannibal et al., 2011; Li et al., 2011; Micale et al., 2011; Paulussen et al., 2011; Banka et al., 2012; Makrythanasis et al., 2013). Most of those patients have *de novo* mutations in *KMT2D*, whereas obvious autosomal dominant inheritance has been identified in only a few familial cases (Ng et al., 2010).

The second causative gene of KS is *KDM6A* (OMIM ^∗^300128), encoding lysine-specific demethylase 6A, which causes X-linked KS subtype 2 when mutated (OMIM #300867). Germline mutations in *KDM6A* are relatively rare, and are responsible for fewer than 5% of patients with KS (Lederer et al., 2012; Miyake et al., 2013; Banka et al., 2015). Most KS patients, especially those with typical facial dysmorphism, carry *KMT2D* mutations (Banka et al., 2012). However, phenotypical variability has been documented in individuals with *KMT2D* mutations, indicating that such mutations can be detected in KS patients with atypical manifestations.

Kabuki syndrome and CHARGE syndrome are distinct congenital disorders, although phenotypic and molecular links between them have been reported previously (Ming et al., 2003; Genevieve et al., 2004; Schulz et al., 2014; Verhagen et al., 2014; Badalato et al., 2017; Butcher et al., 2017). However, to our knowledge, there are no reports of KS cases that have met both Blake and Verloes diagnostic criteria for CHARGE syndrome (Blake et al., 1998; Verloes, 2005). We herein report a patient clinically diagnosed with typical CHARGE syndrome that fulfilled both Blake and Verloes criteria (Blake et al., 1998; Verloes, 2005), but who was genetically diagnosed with atypical KS based on the presence of a *de novo KMT2D* mutation and the absence of pathogenic variation in *CHD7*. This case demonstrates the phenotypic overlap between CHARGE syndrome and KS.

## Case Report

**FIGURE 1:**
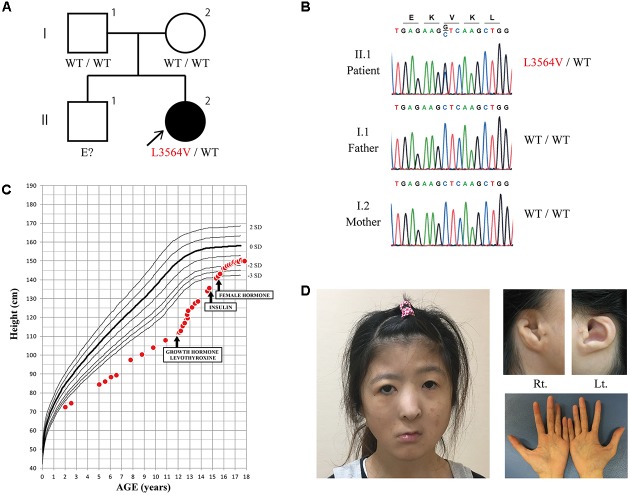
**(A,B)** Family tree and sequence chromatogram. The heterozygous *KMT2D* mutation L3564V was identified in the proband (II.2). The same mutation was not found in her parents (I.1 and I.2), suggesting that it was a *de novo* mutation. **(C)** Patient growth curve. Severe growth failure was improved by the introduction of growth hormone and levothyroxine. Three years after starting growth hormone therapy, insulin therapy was started to treat diabetes associated with insulin insufficiency. **(D)** Photographs of the face, ears, and hands at age 24 years. (Left) The patient had right facial nerve palsy, and a broad and depressed nasal tip. Her long eyelids with eversion of the lateral third of the lower eyelid, which is a typical finding of Kabuki syndrome, are not obvious in the photograph. (Top right) Abnormality of the external ear. Large prominent earlobes, which are a typical finding of Kabuki syndrome, were absent. (Bottom right) No abnormalities of the fingertip pads or the hockey-stick palmar crease were observed.

The 24-year-old Japanese female patient was born to non-consanguineous parents (**Figure 1A**) at 36 weeks and 6 days of gestational age by Cesarean section because of fetal distress. At birth, she had a low body weight (2,100 g) and a short stature (44 cm). She presented with multiple dysmorphic features including choanal atresia, cleft palate, micrognathia, a hypoplastic cupped auricle with atresia of the external auditory meatus, and right facial nerve palsy. Echocardiography revealed no structural abnormalities of the heart, and no coloboma was observed in either eye, together with no abnormalities in the iris, retina, choroid, or optic disk. Chromosome analysis revealed a normal karyotype (46, XX). Mechanical ventilation was required for respiratory distress for 2 weeks, after which an oropharyngeal tube was used to maintain the airways until she was 7 months old. Her choanal atresia was treated surgically at age 1 year. She had tooth hypoplasia with tooth malalignment. She also had entropion of the right upper eyelid, and an epicanthal fold of the left eye that were surgically treated at age 11 years.

**FIGURE 2:**
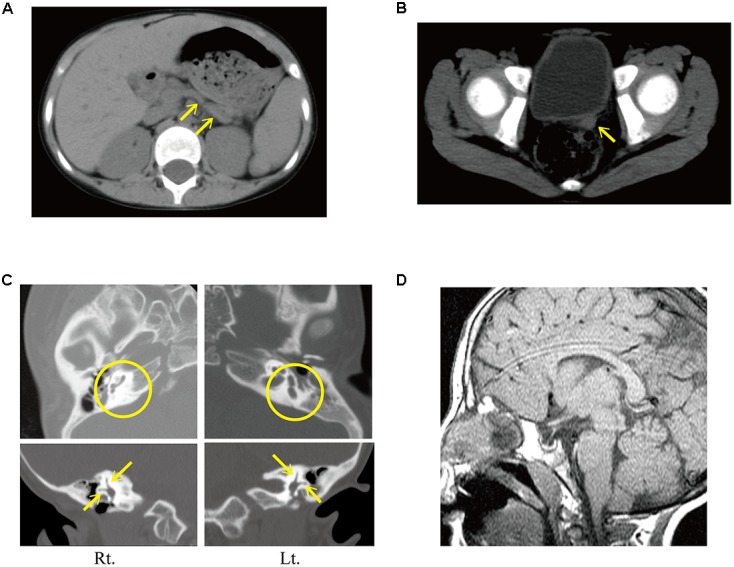
**(A–C)** Computed tomography findings at age 11 years. **(A)** Hypoplasia of the pancreatic body and tail (yellow arrow). **(B)** Uterine hypoplasia (yellow arrow) was identified before estrogen replacement therapy. **(C)** Computed tomography finding of the internal ear. (Upper images) Aplasia of both posterior semicircular canals (yellow circles). (Lower images) The presence of anterior and lateral semicircular canals was confirmed (yellow arrows). **(D)** Magnetic resonance imaging showing empty sella.

She presented at our hospital at age 11 years with severe short stature (111.5 cm, -5.87 standard deviation). At the initial visit, she showed deafness, right facial nerve palsy, cleft palate, malformation of the auricle with atresia of the external auditory meatus, and bilateral hypoplastic nipples. Computed tomography revealed hypoplasia of the pancreatic body and tail (**Figure 2A**), and severe uterine hypoplasia with the presence of both ovaries (**Figure 2B**). It also revealed bilateral atresia of the external auditory meatus, hypoplasia of the right vestibular aqueduct, bilateral hypoplasia of the long limb of incus, and bilateral agenesis of stapes and the posterior semicircular canal (**Figure 2C**). Based on these findings, she was clinically diagnosed with typical CHARGE syndrome.

Laboratory examination revealed primary hypopara thyroidism [calcium 6.8 mg/dL (reference range: 8.6–11.0 mg/dL), phosphorus 7.1 mg/dL (reference range: 2.5–4.5 mg/dL), and intact parathyroid hormone 20.0 pg/mL (reference range: 10–65 pg/mL)], and low serum levels of insulin-like growth factor 1 [14.1 ng/mL (reference range: 175–638 ng/mL)] with suspected mild primary hypothyroidism [free thyroxine 0.9 ng/dL (reference range: 1.1–1.9 ng/dL), free triiodothyronine 3.1 pg/mL (reference range: 2.3–4.7 pg/mL), and thyroid-stimulating hormone (TSH) 5.52μU/mL (reference range: 0.48–4.82 μU/mL)] (Supplementary Table 1). The triple stimulation test (insulin, luteinizing hormone-releasing hormone, and thyrotropin-releasing hormone) revealed complete growth hormone (GH) deficiency (peak GH response to insulin and arginine: 1.6 and 2.1 ng/mL, respectively; cut-off point to define severe GH deficiency: <3 ng/mL), hypogonadotropic hypogonadism (peak luteinizing hormone and follicle-stimulating hormone response: 0.1 and 1.0 mIU/mL, respectively), mild primary hypothyroidism [peak TSH: 44.62 μIU/mL (reference < 35 μIU/mL)], and subclinical hyperprolactinemia [peak prolactin: 110.6 ng/mL (reference < 70 ng/mL)] (Supplementary Table 2).

Magnetic resonance imaging of the brain showed an empty sella (**Figure 2D**). The oral glucose tolerance test revealed borderline diabetes with impaired insulin secretion (Supplementary Table 3). The patient started treatment with levothyroxine, alfacalcidol, and GH. Her growth velocity dramatically improved after starting GH therapy (**Figure 1C**). Seven months after starting GH therapy, the repeated oral glucose tolerance test showed borderline diabetes with impaired insulin secretion [insulinogenic index: 0.09 (reference: 1.34 ± 0.66)] without insulin resistance [Homeostasis Model Assessment insulin Resistance (HOMA-R): 0.2 (reference: < 1.6)] (Supplementary Table 3). Serum C-peptide levels were low [0.4 ng/mL (reference range: 1.1–3.3 ng/mL)], but fasting blood glucose, and glycated hemoglobin (HbA1c) were normal, at 102 mg/dL (reference: <126) and 5.3% (reference range: 4.3–5.8%), respectively. However, about 3 years after starting GH therapy, the patient developed diabetes mellitus. She showed elevated fasting blood glucose (301 mg/dL) and HbA1c (9.9%), low C-peptide levels (1.7 ng/mL), but was negative for anti-glutamic acid decarboxylase antibodies. She therefore started insulin therapy.

She underwent plastic surgery to correct the malformation of the auricle five times from age 14 years. She also started estrogen replacement therapy from age 15 years. The patient is currently 24 years old and has multiple dysmorphic features (**Figure 1D**). She has neither fingertip pads nor hockey-stick palmar creases. Genetic testing was performed at age 23 years. Comprehensive DNA sequencing using the TruSight One sequencing panel (Illumina, San Diego, CA, United States) revealed a novel heterozygous mutation, c.10690 C > G (p.L3564V), in *KMT2D*. The mutation was confirmed by Sanger sequencing (**Figure 1B**). It was absent from both of her parents, suggesting that it was *de novo*, and was not found in the public databases (NCBI, Ensembl, dbSNP, or ExAc). It was predicted to be damaging by PolyPhen-2 (probably damaging) and SIFT (damaging) prediction tools, suggesting that it was not an irrelevant polymorphism. A microarray-based comparative genomic hybridization assay revealed no obvious pathogenic DNA copy number aberrations.

## Materials and Methods

Genomic DNA was extracted from peripheral blood samples using the QIAamp Blood Midi Kit (QIAGEN, Hilden, Germany). We performed trio sequencing using a TruSight One sequencing panel consisting of 4813 genes associated with known Mendelian genetic disorders on a MiSeq instrument (Illumina). Sequence data were analyzed using CLC Genomics Workbench version 8.0 (CLC bio, Aarhus, Denmark). Variants detected by MiSeq were validated by conventional Sanger sequencing. Microarray comparative genomic hybridization was performed with the SurePrint G3 Human CGH Micro-array kit 8 × 60 K, Reference DNA Female, and the SureScan Microarray Scanner (Agilent Technologies, Santa Clara, CA, United States). Results were analyzed by CytoGenomics Software version 4.0 (Agilent).

## Discussion

The diagnostic criteria of CHARGE syndrome are defined by two representative papers (Blake et al., 1998; Verloes, 2005). The current patient met the diagnostic criteria of typical CHARGE syndrome defined by Blake et al. by presenting with three major criteria: choanal atresia, characteristic ear abnormalities and cranial nerve dysfunction, and four minor criteria: developmental delay, growth deficiency, an orofacial cleft and genital hypoplasia (based on the finding of hypogonadotropic hypogonadism). She met the diagnostic criteria of typical CHARGE syndrome defined by Verloes by fulfilling two major signs: choanal atresia and hypoplastic semi-circular canals, and four minor signs: rhombencephalic dysfunction, hypothalamo-hypophyseal dysfunction, abnormalities of the middle and external ear, and mental retardation. The patient was therefore clinically diagnosed with typical CHARGE syndrome. In contrast, she did not show choanal atresia or heart defects which are frequently identified in patients with CHARGE syndrome (Pagon et al., 1981; Blake et al., 1998; Zentner et al., 2010). Moreover, comprehensive genetic analysis identified a *de novo* germline mutation, L3564V, in *KMT2D*, which is a gene associated with KS. No mutations or variants were found in *CDH7*. Therefore, the patient was genetically diagnosed with KS despite presenting with typical symptoms of CHARGE syndrome.

A phenotypic overlap between CHARGE syndrome and KS has been described in previous studies (Ming et al., 2003; Genevieve et al., 2004; Schulz et al., 2014; Verhagen et al., 2014; Badalato et al., 2017; Butcher et al., 2017). For example, coloboma is a major symptom, found in 65–90% of patients with CHARGE syndrome (Blake et al., 1998; Zentner et al., 2010). However, coloboma is also found in patients with KS who show a phenotypic overlap between KS and CHARGE syndrome (Ming et al., 2003). Three cases of genetically confirmed KS with *KMT2D* mutations who also met typical CHARGE diagnostic criteria, as defined by either Blake et al. (Schulz et al., 2014; Verhagen et al., 2014) or Verloes (Patel and Alkuraya, 2015), have been previously reported. A detailed comparision of clinical symptoms is shown in Supplementary Tables 4, 5. In contrast, the current case is the first molecularly diagnosed KS patient who simultaneously met two representative diagnostic criteria of typical CHARGE syndrome as defined by Blake et al. and Verloes. It is therefore considered to be an instructive case that clearly indicates the phenotypic overlap between CHARGE syndrome and KS.

The current case presented with several rare KS symptoms. Cranial nerve dysfunction is a typical symptom defined as a major criterion found in 70–92% of patients with CHARGE syndrome (Byerly and Pauli, 1993; Blake et al., 1998, 2008). Specifically, the involvement of cranial nerves I, VII, VIII, IX, and/or X are frequently observed in patients with CHARGE syndrome (Blake et al., 2008). The current case presented with right facial nerve palsy, which occurs in 32–50% of patients with CHARGE syndrome (Blake et al., 2008), but has only been reported in one previous study of a patient with KS (Iida et al., 2006). Choanal atresia is another typical symptom identified in 50–60% of patients with CHARGE syndrome (Blake et al., 1998). However, it is rarely seen in patients with KS, having been reported in four previous studies (Powell et al., 2003; Teissier et al., 2008; Schulz et al., 2014; Badalato et al., 2017). Among those patients, *KMT2D* mutations were only identified in one familial case (Badalato et al., 2017) of autosomal dominant inheritance associated with the Q3575H mutation in exon 38. Surprisingly, the L3564V mutation of the current patient is located in the same exon and affects an amino acid close to that mutated by Q3575H. Further studies are required to determine whether these two missense mutations affect the development of choanal atresia.

Recent studies revealed the presence of molecular link between CHD7 and KMT2D proteins (Schulz et al., 2014; Butcher et al., 2017). Both CHD7 and KMT2D interact with members of the WAR complex, suggesting that these two molecules function as part of the same chromatin modification machinery (Schulz et al., 2014). Butcher et al. (2017) investigated genome-wide DNA methylation profiles in patients with *CHD7* or *KMT2D* mutations, and found that they showed distinct patterns of epigenetic dysregulation. They also identified common DNA methylation signatures, including a gain of DNA methylation at homeobox A5 (HOXA5), which is shared by the two genetic disorders and may account for some of the clinial overlap between CHARGE syndrome and KS. Therefore, both phenotypic and molecular links are observed in patients with CHARGE syndrome and KS.

## Concluding Remarks

We report an atypical case of KS showing clear phenotypic overlap with CHARGE syndrome. This case highlights the importance of considering a diagnosis of KS even if patients fully meet the diagnostic criteria of typical CHARGE syndrome. Therefore, molecular testing of *KMT2D* should be considered in patients clinically diagnosed with CHARGE syndrome without *CHD7* mutations. It also emphasizes the impact of non-biased exhaustive genetic analysis by next-generation sequencing in the molecular diagnosis of rare congenital disorders with atypical manifestations.

## Ethics Statement

We obtained written informed consent for genomic analysis of the patient and her parents in accordance with the Declaration of Helsinki. The genetic study was approved by the Institutional Review Board of Nagoya City University Graduate School of Medical Sciences (approval no. 130). The mother of the patient provided written informed consent for the publication of the patient’s identifiable information.

## Author Contributions

Patient workup: SS, SO, KH, CT, RK, AU-N, SM, and TO. Genetic analysis: KA and HM. Drafted the manuscript: SS, SO, and MK. Final approval of the version to be published: SS, SO, KA, KH, CT, RK, AU-N, SM, TO, HM, and MK. Agreement to be accountable for all aspects of the work: SS, SO, KA, KH, CT, RK, AU-N, SM, TO, HM, and MK.

## Conflict of Interest Statement

The authors declare that the research was conducted in the absence of any commercial or financial relationships that could be construed as a potential conflict of interest.
