# Supplementary material for: Individual Clinically Diagnosed with CHARGE Syndrome but with a Mutation in KMT2D, a Gene Associated with Kabuki Syndrome: A Case Report
Source: Front Genet. 2017 Dec 11;8:210. doi: 10.3389/fgene.2017.00210 (PMC5732153; doi:10.3389/fgene.2017.00210)
Supplement: Supplementary file 1 [file Table_1.docx]

Supplementary Material

**Individual Clinically Diagnosed with CHARGE Syndrome but with a Mutation in *KMT2D*, a Gene Associated with Kabuki Syndrome: A Case Report**

**Sonoko Sakata^1^, Satoshi Okada^1^*, Kohei Aoyama^2^, Keiichi Hara^3^, Reiko Kagawa^1^, Akari Utsunomiya-Nakamura^1^, Shinichiro Miyagawa^1,4^, Tsutomu Ogata^5^, Haruo Mizuno^2^ and Masao Kobayashi^1^**

*** Correspondence: Satoshi Okada**: [saok969@gmail.com](mailto:saok969@gmail.com)

**SUPPLEMENTARY TABLE 1** Patient biochemical and hormonal data.

| **Parameters** | **Patient** | **Reference range** |
| --- | --- | --- |
| WBC (/μL) | 5740 | 4000–9000 |
| RBC (×10^6^/μL) | 4.32 | 3.83–5.00 |
| Hb (g/dL) | 11.6 | 11.5–15.0 |
| Plt (×10^3^/μL) | 223 | 130–350 |
| AST (U/L) | 36 | 8–40 |
| ALT (U/L) | 15 | 0–40 |
| LDH (U/L) | 341 | 110–225 |
| Na (mEq/L) | 144 | 137–149 |
| K (mEq/L) | 3.9 | 3.7–5.0 |
| Cl (mEq/L) | 102 | 98–109 |
| Ca (mg/dL) | 6.8 | 8.6–11.0 |
| P (mg/dL) | 7.1 | 2.5–4.5 |
| TP (g/dL) | 7.0 | 6.5–8.3 |
| Alb (g/dL) | 4.6 | 3.3–4.7 |
| TCHO (mg/dL) | 166 | 150–230 |
| TG (mg/dL) | 166 | 35–160 |
| TSH (μIU/mL) | 5.52 | 0.48–4.82 |
| FT3 (pg/mL) | 3.1 | 2.3–4.7 |
| FT4 (ng/dL) | 0.9 | 1.1–1.9 |
| GH (ng/mL) | 0.2 | 0.0–4.1 |
| IGF-1 (ng/mL) | 14.1 | 175–638 |
| LH (mIU/mL) | <0.1 | 0.02–0.11 |
| FSH (mIU/mL) | 0.2 | 1.16–3.64 |
| E2 (pg/mL) | 8 | <10 |
| intact PTH(pg/mL) | 20 | 10–65 |

Data were obtained from patients aged 11 years.

**SUPPLEMENTARY TABLE 2** Triple stimulation test and arginine tolerance test.

| Insulin, TRH, LHRH tolerance test | Basal | 30 min | 60 min | 90 min | 120 min |
| --- | --- | --- | --- | --- | --- |
| Glucose (mg/dL) | 81 | 29 | 35 | 37 | 35 |
| GH (ng/mL) | 0.8 | 0.6 | 0.5 | 0.6 | 1.6 |
| Cortisol (μg/dL) | 12.6 | 28.4 | 33.3 | 33.7 | 32.8 |
| ACTH (pg/mL) | 28.5 | 114.0 | 107.6 | 77.9 | 69.9 |
| TSH (μIU/mL) | 7.30 | 44.62 | 30.41 | 26.49 | 20.36 |
| PRL (ng/mL) | 15.8 | 110.6 | 81.7 | 71.5 | 52.4 |
| LH (mIU/mL) | <0.1 | 0.1 | <0.1 | <0.1 | <0.1 |
| FSH (mIU/mL) | 0.3 | 1.0 | 1.0 | 1.0 | 0.9 |
| E2 (pg/mL) | 6 |  |  |  | 8 |
| **Arginine tolerance test** | **Basal** | **30 min** | **60 min** | **90 min** | **120 min** |
| GH (ng/mL) | 0.7 | 1.7 | 1.6 | 2.1 | 2.1 |

Data were obtained from patients aged 11 years.

**SUPPLEMENTARY TABLE 3** Oral glucose tolerance tests.

| **At age 12 years: before GH treatment** | | | | | |
| --- | --- | --- | --- | --- | --- |
| **Glucose tolerance test** | **Basal** | **30 min** | **60 min** | **90 min** | **120 min** |
| Glucose (mg/dL) | 82 |  | 206 |  | 157 |
| Insulin (μU/mL) | <1.0 |  | 9.6 |  | 5.7 |
| **At age 12 years 7 months: after GH treatment** | | | | | |
| **Glucose tolerance test** | **Basal** | **30 min** | **60 min** | **90 min** | **120 min** |
| Glucose (mg/dL) | 102 | 183 | 178 | 148 | 140 |
| Insulin (μU/mL) | 2.0 | 9.2 | 6.1 | 6.0 | 9.4 |

**SUPPLEMENTARY TABLE 4** Diagnostic criteria of typical CHARGE syndrome (Blake KD, et al., 1998).

| **Diagnostic criteria** | **Current case** | **Schulz et al., 2014** | **Verhagen et al., 2014** | **Patel et al., 2014** |
| --- | --- | --- | --- | --- |
| **Major criteria** |  |  |  |  |
| Coloboma | - | N.D. | + | + |
| Choanal atresia | + | + | - | - |
| Cranial nerve dysfunction | + | N.D. | + | - |
| Characteristic ear abnormalities | + | N.D. | + | - |
| **Minor criteria** |  |  |  |  |
| Genital hypoplasia | + | + | + | - |
| Developmental delay | + | + | + | + |
| Cardiovascular malformations | - | + | + | + |
| Growth deficiency | + | N.D. | + | + |
| Orofacial cleft | + | - | - | + |
| Tracheoesophageal fistula | - | - | - | - |
| Distinctive facial features | + | N.D. | - | + |
| **Diagnostic criteria** | Definite (3 major, 5 minor) | Definite (3 major, 3 minor)* | Definite (3 major, 4 minor)** | Probable/possible (1 major, 5 minor)** |

[Diagnostic criteria]

Definite CHARGE: 4 major or 3 major and 3 minor criteria

Probable/possible CHARGE: 1 or 2 major and several minor criteria

N.D., not described

* Diagnostic criteria decided and described in the manuscript

** Diagnostic criteria evaluated from the symptoms described in the manuscript

**SUPPLEMENTARY TABLE 5** Diagnostic criteria of typical CHARGE syndrome (Verloes et al., 2015).

| **Diagnostic criteria** | **Current case** | **Schulz et al., 2014** | **Verhagen et al., 2014** | **Patel et al., 2014** |
| --- | --- | --- | --- | --- |
| **Major criteria** |  |  |  |  |
| Coloboma | - | N.D. | + | + |
| Choanal atresia | + | + | - | - |
| Hypoplastic semi-circular canals | + | N.D. | - | + |
| **Minor criteria** |  |  |  |  |
| Rhombencephalic dysfunction | + | - | + | - |
| Hypothalamo-hypophyseal dysfunction | + | N.D. | + | - |
| Abnormal middle or external ear | + | N.D. | + | + |
| Malformation of mediastinal organs | - | + | + | + |
| Mental retardation | + | + | + | + |
| **Diagnostic criteria** | Typical (2 major, 3 minor) | Atypical (1 major, 3 minor)* | Atypical (1 major, 5 minor)** | Typical (2 major, 3 minor)** |

[Diagnostic criteria]

Typical CHARGE: 3 major or 2 major and 2 minor criteria

Partial/incomplete CHARGE: 2 major and 1 minor criteria

Atypical CHARGE: 2 major, or 1 major and 3 minor criteria

N.D., not described

* Diagnostic criteria decided and described in the manuscript

** Diagnostic criteria evaluated from the symptoms described in the manuscript
